# Screening red foxes (*Vulpes vulpes*) for possible viral causes of encephalitis

**DOI:** 10.1186/s12985-016-0608-1

**Published:** 2016-09-02

**Authors:** Manon Bourg, Daniel Nobach, Sibylle Herzog, Hildburg Lange-Herbst, Anne Nesseler, Hans-Peter Hamann, Sabrina Becker, Dirk Höper, Bernd Hoffmann, Markus Eickmann, Christiane Herden

**Affiliations:** 1 Institute of Veterinary Pathology, Justus-Liebig-University, Giessen, Germany; 2 Institute of Virology, Justus-Liebig-University, Giessen, Germany; 3 The Hessian State Laboratory, Giessen, Germany; 4 Friedrich-Loeffler-Institute, Greifswald, Germany; 5 Institute of Virology, Philipps-University, Marburg, Germany

**Keywords:** Borna disease, BoDV-1, Red fox, Non-suppurative encephalitis, Indirect immunofluorescence test, Pan-bornavirus-RT-PCR, NGS, Canine distemper, Germany

## Abstract

**Background:**

Next to various known infectious and non-infectious causes, the aetiology of non-suppurative encephalitis in red foxes (*Vulpes vulpes*) often remains unclear. Known causes in foxes imply rabies, canine distemper, toxoplasmosis, Aujeszky’s disease, as well as parvovirus, adenovirus, circovirus and flavivirus infections. In this study, particular attention was paid on bornaviruses, since red foxes are predators of bicoloured white-toothed shrews, a reservoir of Borna disease virus 1 (BoDV-1). In addition, foxes are known to be highly susceptible for viruses of the order *Mononegavirales*.

**Methods:**

Analyses for the presence of anti-BoDV-1 antibodies, BoDV-1-RNA and antigen were performed on 225 blood and 59 brain samples, from a total of 232 red foxes. Foxes originated from BoDV-1 endemic and non-endemic German areas. Additional investigations for the presence of rabies, canine distemper, toxoplasmosis, Aujeszky’s disease, parvovirus, adenovirus and flavivirus infections were carried out on 16 red foxes with non-suppurative (meningo-) encephalitis. A metagenomic analysis was used on three representative brain samples displaying encephalitis.

**Results:**

Among 225 foxes, 37 displayed anti-BoDV-1 antibodies with titres ranging between 1:40 and 1:2560, regardless of geographic origin. In 6 out of 16 foxes with encephalitis, canine distemper virus was detected. No evidence of any of the other investigated agents was found in the 16 fox brains with encephalitis. Metagenomics revealed no infectious agents, except for one already known canine distemper case.

**Conclusion:**

Red foxes can exhibit BoDV-1 specific antibodies without association with geographic origin or encephalitis due to bornavirus infection. The encephalitis pattern was highly conspicuous for a viral infection, but remained unclear in 10 out of 16 foxes. Thus, presently unknown infectious and non-infectious causes need to be considered and further investigated, especially since foxes also tend to occur in human proximity.

## Background

Red foxes (*Vulpes vulpes*) are known to suffer regularly from non-suppurative encephalitis. However, the underlying cause remains unclear in a remarkable number of cases, even though several infectious and non-infectious causes for encephalitis in canines are known to date. This is a known problem in other canines, felines and cattle as well [1–7]. Infectious or non-infectious causes can induce neuroinflammation. Non-infectious causes are, for example, post-traumatic processes, autoimmune diseases, genetic and metabolic disorders or damages induced by toxic agents. In terms of infectious encephalitis, suppurative or granulomatous inflammation are usually caused by bacterial or mycotic infections. Fibrinous, haemorrhagic and mixed inflammation also tend to occur due to bacterial or viral infections. However, non-suppurative encephalitis is most commonly of viral origin [8] and characterised by perivascular mononuclear cuffing, glial proliferation and, in many cases, neuronal degeneration and satellitosis [6, 8, 9].

It is known that red foxes play a role in spreading pathogens which is of importance due to their adaptation to human environments and to their invasion into urban life. Thus, viral pathogens causing encephalitis with unclear pathogenic and potential zoonotic potential could represent a serious threat for humans and animals alike [10, 11]. Viral agents known to induce (meningo-) encephalitis in canines comprise *Rhabdoviridae* [12], *Paramyxoviridae* [13, 14], *Herpesviridae* [15], *Parvoviridae* [5, 16], *Flaviviridae* [5], *Arboviridae* [17], *Bornaviridae* [18] and *Circoviridae* [19]. Protozoal and bacterial agents, like *Toxoplasma gondii*, *Neospora caninum*, *Encephalitozoon cuniculi* and *Listeria monocytogenes* can occasionally induce widespread non-suppurative meningoencephalitis also in canines [8, 20, 21]. Next to infectious agents, non-infectious causes have been considered as well to explain unclear encephalitis in canines [6, 22].

A causative pathogen of non-suppurative encephalitis in mammals is Borna disease virus 1 (BoDV-1), which belongs to the family of *Bornaviridae* within the order of *Mononegavirales*. Infection with BoDV-1 leads to Borna disease (BD), a lethal neurological disease in accidental hosts such as horses and sheep [23]. Numerous studies have shown other mammals, including farm (cattle, goats) and companion animals (dogs), to occasionally succumb to natural BD [23, 24]. BD was also reported in free ranging animals [23, 24]. Moreover, canines, especially red foxes are highly susceptible for viruses of the order of *Mononegavirales* (*Rhabdoviridae*, *Paramyxoviridae*), which can cause fatal neurological diseases. In addition, foxes are predators of the bicoloured white-toothed shrew *Crocidura leucodon* (*C. leucodon*), which represent a reservoir for BoDV-1 in Germany and Switzerland [25–29]. As these shrews shed the virus via various routes – including saliva, urine, faeces and skin – foxes have to be exposed to high amounts of infectious BoDV-1 during predation [29].

Knowledge on bornaviruses has expanded remarkably over the last years and new avian, reptile and mammalian bornaviruses have been found [30]. Recently, a new mammalian bornavirus (variegated squirrel 1 bornavirus, VSBV-1), found in variegated squirrels (*Sciurus variegatoides*) in Germany caused fatal encephalitis in three squirrel breeders and represented the first bornavirus with proven lethal zoonotic capacity [31]. Evidence of a highly pathogenic and zoonotic bornavirus places new demands in public health security, especially since the source of the new virus remains unknown so far. Therefore, it is vital to increase knowledge around the potential association of encephalitis cases of unknown origin in foxes with such newly discovered agents.

Reports on BoDV-1 infection in canines are sparse. In a study comprising foxes from France, viral BoDV-1 RNA was detected in 6 among 59 brains by nested PCR. These positive results could, however, not be reproduced by others [32]. In another study from Austria, BoDV-1 infection in a 2-year-old female dog with a non-suppurative meningoencephalitis from Vorarlberg, an endemic area in Austria, was confirmed by detection of viral antigen and RNA by immunohistochemistry, in situ hybridization and nested PCR procedures, respectively. [18]. Another canine case was described in Japan, where a 3-year-old dog with a severe neurological disorder was diagnosed with clinical BD but no BoDV-1 specific sequences were published [33]. Besides, in Germany, about 10 % of the dogs display anti-BoDV-1 antibodies (personal communication S. Herzog). To our knowledge, there are no other studies on the prevalence of anti-BoDV-1 antibodies in canines. In another small carnivore, the cat, BoDV-1 is suspected to cause a severe neurological disease called “staggering disease” [34]. In other studies, BoDV-1 RNA and/or antigen was not found in respective cat material [35]. Considering the new findings in bornavirus research, reinvestigation of cat material by novel metagenomics approaches might be an option.

To sum up, red foxes can play a role in spreading pathogens (*Mononegavirales,* among others) and can have contact to reservoir species such as BoDV-1 shedding shrews due to their lifestyle [10]. However, the outcome of contact exposure with viruses such as BoDV-1 in foxes remains unknown. There is need for examination on whether foxes can represent any kind of clinically inconspicuous reservoir, spill over host or accidental host with non-suppurative meningoencephalitis. The occurrence of a new zoonotic bornavirus with unknown reservoir and origin underlines the need for further epidemiological surveys on bornavirus infection in potential contact animals. Beside this, it is important to assess so far unknown infectious and non-infectious causes for encephalitis in red foxes, a need for which this study accounts as well. As foxes are a widespread wild life species in Central Europe, also in urban areas, knowledge on the aetiology of unknown non-suppurative encephalitis in this species is of high importance.

## Methods

### Samples from red foxes

Brain and blood samples from wild red foxes (*Vulpes vulpes*) were collected from 2013 to 2014 in three federal states (Bavaria, Baden-Wuerttemberg and Hesse) in Germany, in collaboration with State Veterinary Institutes and private hunters, in endemic and non-endemic regions for BoDV-1. Brain samples, initially intended for rabies virus investigation, comprised parts of the hippocampus, thalamus and cerebral cortex. Private hunters were encouraged to collect blood samples in the hunting season immediately after the foxes’ death. The State Veterinary Institutes of Aulendorf (Staatliches Tierärztliches Untersuchungsamt Aulendorf, STUA), Freiburg (Chemisches und Veterinäruntersuchungsamt Freiburg, CVUA) and Giessen (Landesbetrieb Hessisches Landeslabor, LHL) and private hunters in Bavaria, Baden-Wuerttemberg and Hesse collected 59 brain and 225 blood samples from 232 red foxes (*Vulpes vulpes*). Additionally, the LHL Giessen provided formalin fixed tissue sections from three red foxes with encephalitis of unknown origin (#41, #42, and #43). Blood samples were collected in serum separating tubes (BD Vacutainer®, Becton, Dickinson and Company, USA and Monovettes®, Sarstedt, Germany). After visual control of complete coagulation, the serum separating tubes were deep-frozen. Overall, sample collection included road kill and foxes that were found dead. Information about age, sex and clinical signs was requested.

### Serology for the detection of anti-BoDV-1 antibodies

An indirect immunofluorescence test (IIFT) detected BoDV-1-specific serum antibodies as described elsewhere [36, 37]. To sum up, after thawing, the 225 blood samples were centrifuged at 2500 rpm for 10 min to obtain serum. Several dilutions of sera were incubated on slides with acetone-fixed MDCK-cells (Madin-Darby canine kidney), persistently infected with BDV-H1766 (horse strain). After incubation for 30 min at 37 °C, cells were exposed for 30 min with a fluorescein isothiocyanate (FITC)-conjugated rabbit anti-dog IgG (Dianova, Germany), again at 37 °C. To confirm the specificity of the anti-BoDV-1 antibodies, sera of 8 out of 37 selected seropositive foxes underwent further western blot analysis as described by Richt et al. [38].

### Histology and immunohistochemistry for the detection of BoDV-1 antigen

Tissue sections of 4 μm were routinely stained with haematoxylin and eosin and evaluated for the presence of inflammatory or degenerative lesions.

For immunohistochemistry (IHC) detecting BoDV-1 antigen, the standard avidin-biotin-peroxidase complex (ABC) method was used, applying a monoclonal anti-BoDV-1 antibody (Bo18) and a polyclonal anti-BoDV-1 antibody (p24), as described elsewhere [39–41]. For further detail on antigen retrieval methods, dilution and the origin of the antibodies, see Table 1. Positive controls consisted of mammals infected with the respective agent, mostly samples from infected dogs. Brain tissue of a naturally infected horse was used as a control for BoDV-1 infection.

**Table 1:** Antibodies used for the detection of infectious agents by immunohistochemistry

Infectious agent	Abbreviation	Primary antibody	Dilution	Antigen retrieval Method	Origin of primary antibody
Borna disease virus	BoDV-1	monoclonal mouse-anti-p38 (Bo18)	1:500^a^	None	Dr. Herzog, Giessen, Germany
Borna disease virus	BoDV-1	polyclonal rabbit- anti-p24 (p24)	1:2000^b^	None	Dr. Richt, Kansas, USA
Canine distemper virus	CDV	monoclonal mouse-anti-CDV	1:6000^a^	None	Dr. Örwall, Huddinge, Sweden
Porcine herpesvirus-1	SHV-1	polyclonal rabbit-anti-SHV-1	1:2000^b^	None	Dr. Eskens, Giessen, Germany
Canine adenovirus 1	CAV-1	monoclonal anti-canine adenovirus (CAV4-1A)	1:100^a^	Protease-induced epitope retrieval	Custom Monoclonals International Corp., USA
Canine parvovirus	CPV	monoclonal anti-parvovirus (CPV1-2A1)	1:400^a^	Protease-induced epitope retrieval	Custom Monoclonals International Corp., USA
*Toxoplasma gondii*	None	polyclonal rabbit-anti-toxoplasma gondii	1:800^b^	Protease-induced epitope retrieval	DAKO, Hamburg, Germany

^a^ in tris-buffered saline (TBS) containing bovine serum albumin (BSA) 1 %, ^b^ in TBS containing 20 % swine serum

### RT-PCR assays for the detection of BoDV-1 RNA

RNA extraction and real-time reverse transcription polymerase chain reaction were performed using the commercially available kits QIAsymphony RNA Kit and OneStep RT-PCR kit (both Qiagen, Germany). The commercially available kit RNeasy FFPE Kit (Qiagen) was used for RNA isolation from formalin-fixed material. Amplification of BoDV-1 RNA was carried out by one-step real time RT-PCR approaches [42]. For questionable results from RT-PCR a nested PCR was applied according to existing protocols [43].

As baseline, all fox brain samples were checked for the suitability of the cDNA by a GAPDH-PCR (glyceraldehyde-3-phosphate dehydrogenase-PCR). (Details of the protocols are available upon request).

### Pan-bornavirus-RT-PCR

Additionally, a broad-range-bornavirus-RT-PCR was designed to cover all known mammalian and most of the avian bornaviruses. RNA was extracted using the QIAmp Viral Mini Kit (Qiagen) according to the manufacturer’s instructions. Conserved nucleotide sequences of all available bornaviruses were used for primer design. Samples were analysed for the presence of bornaviruses, by amplifying a 61-bp fragment of the P-gene and the end of the X-gene using the following primer set: forward primer Borna-2048-F: 5′-CGC GAC CMT CGA GYC TRG T-3′ and reverse primer Borna-2118-R: 5′-GAC ARC TGY TCC CTT CCK GT-3′ (Biomers.net, Germany). The RT-reaction was performed with the QuantiTect Reverse Transcription-Kit (Qiagen) employing 1000 ng RNA and the manufacturer’s primer-mix followed by PCR with the PCR Multiplex PCR kit (Qiagen) according to manufacturer’s instructions and a primer concentration of 0,25 pg/μl. Cycling conditions consisted in an initial activation of the Taq Polymerase at 95 °C for 15 min, followed by 45 cycles of denaturation at 94 °C for 30 s, annealing at 60 °C for 30 s and extension at 72 °C for 30 s, finally for 10 min. Amplified PCR-products were separated on a 2 % agarose gel with 3,6 μl/100 ml Midori-Green (Biozym, Germany) and a standard length (pUC 8 Mix Marker, Thermo Fischer Scientific, USA). The pan-bornavirus-RT-PCR constantly detected BoDV-1, parrot bornavirus 2 and 4 (PaBV-2 and PaBV-4) and VSBV-1. The sensitivity of the pan-bornavirus-RT-PCR protocol was determined by amplification of serial dilutions of PaBV-2 in purified fox control RNA (700 ng/μl) and RNase-free water, followed by agarose gel electrophoresis. The detection limit was 0.01 ng/μl PaBV-2 in 700 ng/μl fox RNA.

### Methods used for the detection of other pathogens

The State Veterinary Institutes screened all brain samples for the presence of rabies virus, using an immunofluorescence test (IFT) as a standard method recommended by the WHO and OIE. Immunohistochemistry was used for the presence of antigens of canine distemper virus (CDV), porcine herpesvirus 1 (SHV-1), canine adenovirus 1 (CAV-1), canine parvovirus (CPV) and of *Toxoplasma gondii* (Table 1). The State Veterinary Institutes Giessen, Freiburg and Aulendorf routinely conducted RT-PCR assays for CDV RNA according to established protocols [44]. At the Institute of Veterinary Pathology in Giessen, a pan-flavivirus-RT-PCR protocol [45] and an adopted PCR assay for the amplification of CPV [46] was used.

### Metagenomics, next generation sequencing (NGS)

Representative brain samples of three red foxes with high anti-BoDV-1 antibody titres and encephalitis were sequenced, using a MiSeq instrument [Illumina] as described before [31]. Sequence analysis with RIEMS assembled the sequences [47].

### Statistical analysis

For comparison of detection of seropositive foxes in endemic and non-endemic administrative districts, a hypothesis test, the two-proportion z-test, with a significance level equal to 0.05 was used.

## Results

### Origin of fox samples

**Fig. 1:**
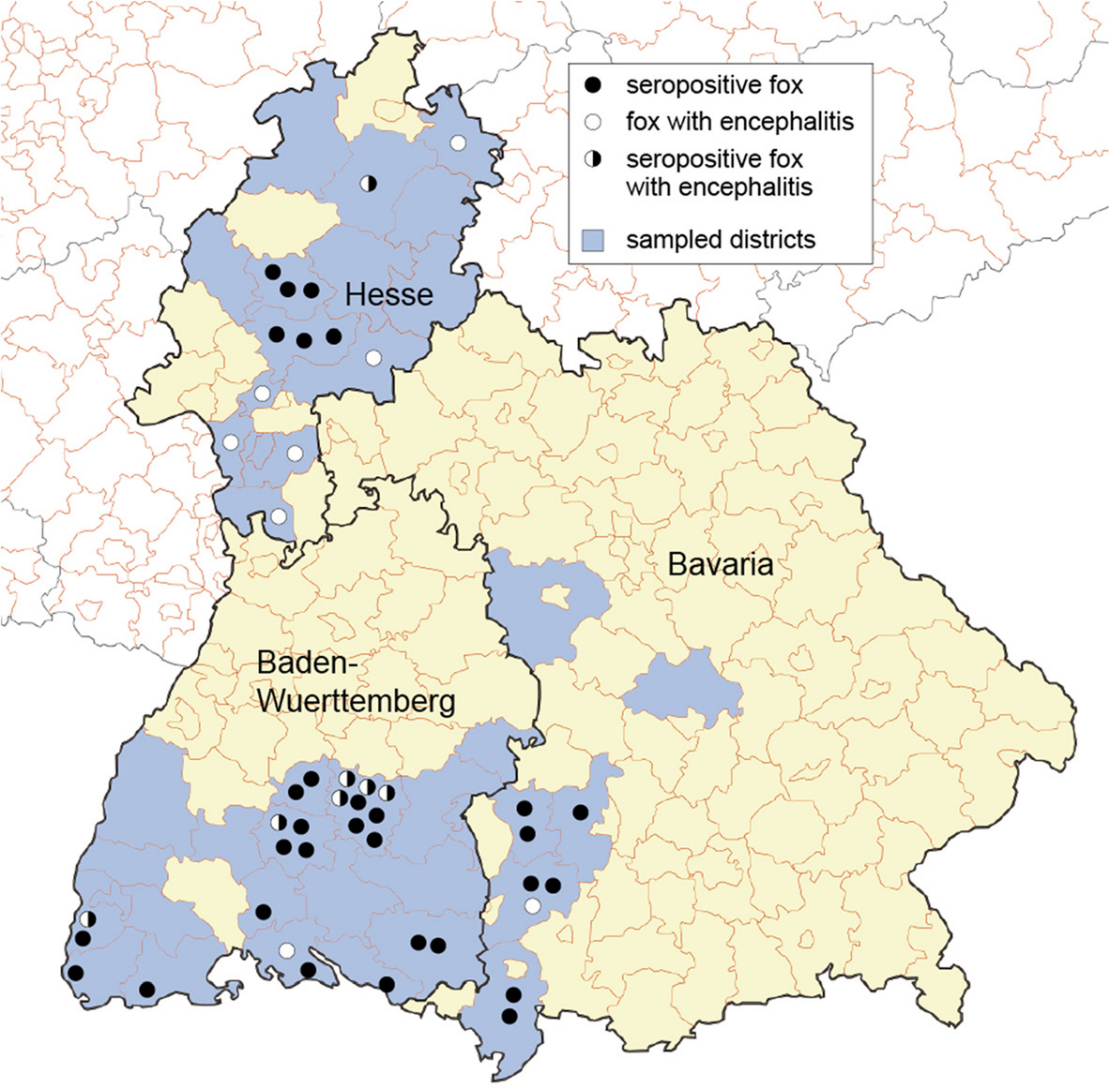
Distribution of seropositive foxes and foxes with encephalitis in Bavaria, Baden-Wuerttemberg and Hesse

The State Veterinary Institutes and private hunters in Bavaria, Baden-Wuerttemberg and Hesse collected samples from 232 red foxes (59 brain, 225 blood samples) and formalin fixed tissue sections from three red foxes with encephalitis of unknown origin (#41, #42, and #43). Private hunters provided 81 frozen blood samples from 40 hunting districts in 10 administrative districts in Germany. Sample collection was scattered geographically, depending on the location of the State Veterinary Institutes and the private hunters involved in the study including samples from known endemic and non-endemic areas. Among 232 carnivores, 64 small carnivores originated from Bavaria (Swabia, Upper Bavaria, and Middle Franconia), 132 from Baden-Wuerttemberg (Tubingen and Freiburg) and 36 from Hesse (Kassel, Giessen and Darmstadt). See Fig. 1 for further details on the sampled districts.

### Serology for detection of anti-BoDV-1 antibodies

An indirect immunofluorescence test (IIFT) was carried out for 225/232 red foxes. Among 225 foxes, 37 exhibited anti-BoDV-1 antibodies, representing a prevalence rate of 16.4 % in the investigated districts in Germany. Sera with anti-BoDV-1 antibodies caused a brilliant granular fluorescence in the nucleus of BoDV-1 infected MDCK cells. In Bavaria, 7/63 foxes displayed anti-BoDV-1 antibodies, in Baden-Wuerttemberg 23/131 and in Hesse 7/31. For an overview of results for seropositive foxes in endemic and non-endemic districts see Table 2 and Fig. 1. Statistical analysis showed the *p*-value (0.36) to be higher than the significance level (0.05), which is why the null hypothesis cannot be rejected. No statistically significant difference has been found between the detection of seropositive foxes in endemic and non-endemic areas in the investigated areas in Germany. Serum antibody titres in foxes ranged from 1:40 to 1:2560 (median 1:160). The red fox #21 with a titre of 1:2560 was an adult female from the administrative district of Reutlingen, an endemic region in Baden-Wuerttemberg. Among the 37 seropositive foxes, hunters observed no clinical symptoms in 23 canines. However, eight foxes showed signs of illness: two had a mange dermatitis and six were emaciated. No information on clinical symptoms was available for six foxes. For further details on age, gender and clinical symptoms of seropositive foxes, see Table 3.

**Table 2:** Origin of fox blood samples and geographical distribution of seropositive foxes

	Total number of fox blood samples used for IIFT			Seropositive foxes		
Administrative district	Total number of blood samples	Total from endemic regions	Total from non-endemic regions	Total number of seropositive foxes	Total number of seropositives from endemic regions	Total number of seropositives from non-endemic regions
Bavaria	63	32	31	7	5	2
Baden-Wuerttemberg	131	56	75	23	12	11
Hesse	31	0	31	7	0	7
Germany	225	88	137	37	17	20

**Table 3:** Further investigations for the presence of BoDV-1 in seropositive foxes or foxes with encephalitis

Sample	Administrative district	Age	Sex m/f	Clinical signs	Histology	IIFT	IHC Bo18	IHC p24	BoDV-1 PCR	Pan borna virus PCR
Bavaria										
1	LKR Günzburg	adult	m	no signs	n.d	1:160	n.d.	n.d.	n.d.	n.d.
2	LKR Günzburg	adult	m	no signs	n.d	1:640	n.d.	n.d.	n.d.	n.d.
3	LKR Augsburg	juvenile	n.d.	no signs	n.d	1:40	n.d.	n.d.	n.d.	n.d.
4	LKR Unterallgäu	juvenile	n.d.	no signs	n.d	1:160	n.d.	n.d.	n.d.	n.d.
5	LKR Unterallgäu	juvenile	n.d.	no signs	-	1:80	−	−	−	−
6	LKR Unterallgäu	adult	f	found dead	encephalitis^a^	<1:10	−	−	−	−
7	LKR Oberallgäu	adult	m	n.d.	n.d	1:640	n.d.	n.d.	n.d.	n.d.
8	LKR Oberallgäu	adult	n.d.	no signs	n.d	1:160	n.d.	n.d.	n.d.	n.d.
Baden-Wuerttemberg										
9	LKR Waldshut	juvenile	m	road kill	−	1:160	−	−	n.d.	n.d.
10	LKR Breisgau-Hochschwarzwald	adult	f	road kill	−	1:160	−	−	−	−
11	LKR Breisgau-Hochschwarzwald	juvenile	m	found dead	meningoencephalitis	1:160	−	−	−	−
12	LKR Lörrach	adult	m	mange	−	1:40	−	−	−	−
13	LKR Tuttlingen	juvenile	n.d.	no signs	−	1:640	−	−	−	−
14	LKR Tübingen	adult	m	emaciation	−	1:160	−	n.d.	−	−
15	LKR Tübingen	adult	m	no signs	−	1:40	−	−	−	−
16	Zollernalbkreis	n.d.	n.d.	no signs	encephalitis^a^	1:160	−	−	−	−
17	Zollernalbkreis	adult	m	no signs	n.d	1:640	n.d.	n.d.	n.d.	n.d.
18	Zollernalbkreis	juvenile	n.d.	no signs	n.d	1:160	n.d.	n.d.	n.d.	n.d.
19	Zollernalbkreis	n.d.	n.d.	no signs	n.d	1:640	n.d.	n.d.	n.d.	n.d.
20	LKR Reutlingen	adult	m	no signs	−	1:160	−	−	−	−
21	LKR Reutlingen	adult	f	no signs	encephalitis^a^	1:2560	−	−	−	−
22	LKR Reutlingen	adult	f	no signs	encephalitis^a^	1:160	−	−	−	−
23	LKR Reutlingen	adult	m	no signs	−	1:160	−	−	−	−
24	LKR Reutlingen	n.d.	n.d.	no signs	−	1:160	−	n.d.	−	−
25	LKR Reutlingen	juvenile	m	emaciation	−	1:160	−	n.d.	−	−
26	LKR Reutlingen	adult	m	no signs	encephalitis^a^	1:160	−	−	−	−
27	LKR Reutlingen	adult	f	emaciation	encephalitis^a^	1:160	−	−	−	−
28	LKR Ravensburg	juvenile	m	emaciation	−	1:640	−	−	−	−
29	LKR Ravensburg	adult	f	emaciation	−	1:160	−	n.d.	−	−
30	LKR Konstanz	n.d.	n.d.	n.d.	−	1:640	−	−	−	−
31	LKR Konstanz	adult	m	no signs	meningoencephalitis	<1:10	−	−	−	−
32	Bodenseekreis	adult	m	emaciation	−	1:640	−	−	−	−
33	n.d. Baden-Wuerttemberg	n.d.	n.d.	emaciation	encephalitis^a^	<1:10	−	−	−	−
Hesse										
34	LKR Gießen	adult	n.d.	no signs	n.d	1:40	n.d.	n.d.	n.d.	n.d.
35	LKR Gießen	adult	n.d.	no signs	n.d	1:160	n.d.	n.d.	n.d.	n.d.
36	LKR Gießen	juvenile	n.d.	found dead	−	1:40	−	−	−	−
37	Wetteraukreis	n.d.	n.d.	no signs	−	1:160	−	−	−	−
38	Wetteraukreis	n.d.	n.d.	no signs	n.d	1:40	n.d.	n.d.	n.d.	n.d.
39	Wetteraukreis	n.d.	n.d.	no signs	−	1:160	−	−	−	−
40	Schwalm-Eder-Kreis	juvenile	n.d.	mange	meningoencephalitis	1:160	−	−	−	−
41	LKR Groß-Gerau	adult	n.d.	no signs	meningoencephalitis	n.d	−	−	−	−
42	LKR Bergstraße	adult	n.d.	found dead	meningoencephalitis	n.d	−	−	n.d.	n.d.
43	Main-Kinzig-Kreis	adult	n.d.	no signs	meningoencephalitis	n.d	−	−	−	−
44	Stadt Frankfurt a. M.	adult	n.d.	found dead	meningoencephalitis	n.d	−	−	−	−
45	LKR Darmstadt-Dieburg	adult	n.d.	no signs	encephalitis^a^	n.d	−	−	−	−
46	Werra-Meißner-Kreis	adult	n.d.	no signs	encephalitis^a^	<1:10	−	−	−	−

+ positive test result, −negative test result, n.d. not determined, ^a^no meninges available

### Histology and immunohistochemistry for the detection of BoDV-1 antigen

**Fig. 2:**
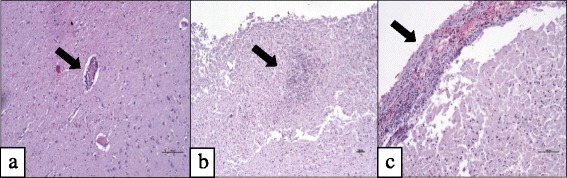
Meningoencephalitis in red foxes. **a** Mononuclear perivascular cuffs in the brain. **b** Gliosis. **c** Non-suppurative meningitis

By histology, 59 brains from red foxes were analysed. Mild to moderate autolytic changes and freezing artefacts were obvious in all cases. In total, 16/59 foxes displayed a non-suppurative encephalitis (Fig. 2). Exactly seven of the 16 foxes had a non-suppurative mononuclear meningoencephalitis and the other nine displayed a non-suppurative mononuclear encephalitis. In the 9/16 cases with encephalitis only, no meninges were present. Histopathology revealed perivascular mononuclear infiltration and microglia activation. Among 16 red foxes with encephalitis, seven displayed anti-BoDV-1 antibodies, four were seronegative and for five, no blood samples were available. The hunters noticed no signs of a neurological disorder in the foxes with encephalitis. However, no information on clinical symptoms was available for four foxes, as they were found dead. For further information on origin, age, gender and clinical symptoms of foxes with encephalitis see Table 3 and Fig. 1.

No evidence of BoDV-1 antigen was found in the foxes by immunohistochemistry. There was no detection of the viral nucleoprotein in 59 foxes using a monoclonal antibody. Among 34 foxes tested with the polyclonal serum for the detection of the viral phosphoprotein, 28 were negative and six fox brains could not be considered further, due to autolytic changes.

### RT-PCR assays for the detection of BoDV-1 RNA

All seropositive (for BoDV-1 antibodies) foxes as well as foxes with (meningo-) encephalitis were analysed for the presence of BoDV-1 RNA in the brain by real time RT-PCR. Among 47 foxes, 33 brain samples meeting the previously mentioned conditions were tested. Brain sample #42 failed in the DNA quality check, since GAPDH could not be amplified and was therefore not further analysed. All 32 foxes remained negative for BoDV-1 RNA, except for three cases. Foxes #40, #41 and #43 exhibited questionable results (ct values near threshold) by the applied real time RT-PCR assay. However, no BoDV-1 RNA could be amplified in the subsequent nested PCR. BoDV-1 RNA was also not found in any of the 32 fox brains using the pan-bornavirus-RT-PCR (fox #42 is excluded).

### Immunohistochemistry for detection of other viral and parasitic agents

**Fig. 3:**
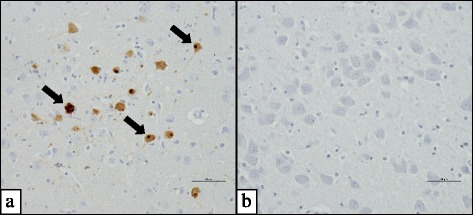
Detection of CDV antigen by immunohistochemistry in a red fox. **a** Canine distemper virus antigen was detected in fox #33 by immunohistochemical staining. **b** Negative control

Among 16 red foxes with non-suppurative encephalitis, six were positive for canine distemper virus (CDV) by RT-PCR (#16, #21, #22, #26, #27, #33) but CDV antigen was detected only in 5/6 red foxes (Fig. 3) (#21 was negative). All CDV-positive foxes originated from Baden-Wuerttemberg, see Table 4 for further details. Among six red foxes with CDV infection, five were also positive for anti-BoDV-1 antibodies (#16, #21, #22, #26, and #27). Brain sample #42 failed in the DNA quality check and was not used for PCR assays. The State Veterinary Institutes provided negative results for rabies virus for all 16 foxes with unclear encephalitis. All foxes with encephalitis remained negative for antigens of parvovirus, adenovirus, porcine herpesvirus 1 and *Toxoplasma gondii*. Furthermore, infection with West Nile virus (WNV), tick-borne encephalitis virus (TBEV) or any other flavivirus could not be confirmed due to 15/15 negative pan-flavivirus-RT-PCR results (fox #42 is excluded). Thus, the cause of the non-suppurative (meningo-) encephalitis remains unclear in 10/16 red foxes. See Table 4 for an overview of pathogens tested.

**Table 4:** Investigation on causes for fox encephalitis other than BoDV-1

		CDV		Rabiesvirus	SHV-1	CAV-1	Flaviviridae	CPV		*T. gondii*
Sample	Administrative district	PCR	IHC	IFT	IHC	IHC	Pan flavivirus PCR	IHC	PCR	IHC
Bavaria										
6	LKR Unterallgäu	−	−	−	−	−	−	−	−	−
Baden-Wuerttemberg										
11	LKR Breisgau-Hochschwarzwald	−	−	−	−	−	−	−	−	−
16	Zollernalbkreis	+	+	−	−	−	−	−	−	−
21	LKR Reutlingen	+	−	−	−	−	−	−	−	−
22	LKR Reutlingen	+	+	−	−	−	−	−	−	−
26	LKR Reutlingen	+	+	−	−	−	−	−	−	−
27	LKR Reutlingen	+	+	−	−	−	−	−	−	−
31	LKR Konstanz	−	−	−	−	−	−	−	−	−
33	n.d. Baden-Wuerttemberg	+	+	−	−	−	−	−	−	−
Hesse										
40	Schwalm-Eder-Kreis	−	−	−	−	−	−	−	−	−
41	LKR Groß-Gerau	−	−	−	−	−	−	−	−	−
42	LKR Bergstraße	n.d.	−	−	−	−	n.d.	−	n.d.	−
43	Main-Kinzig-Kreis	−	−	−	−	−	−	−	−	−
44	Stadt Frankfurt a. M.	−	−	−	−	−	−	−	−	−
45	LKR Darmstadt-Dieburg	−	−	−	−	−	−	−	−	−
46	Werra-Meißner-Kreis	−	−	−	−	−	−	−	−	−

CDV canine distemper virus; SHV-1 porcine herpesvirus 1, CAV-1 canine adenovirus 1, CPV canine parvovirus

+ positive test result, −negative test result, *n.d* not determined

### Metagenomics, next generation sequencing (NGS)

Among 16 foxes with (meningo-) encephalitis, three fox brains (#11, #21, #40) were further analysed by a metagenomic analysis. All three foxes displayed high anti-BoDV-1 antibody titres (1:160–1:2650) and a mild to moderate non-suppurative (meningo-) encephalitis. Foxes #11 and #21 originated from Baden-Wuerttemberg and fox #40 from Hesse. Fox #21 originated from the Swabian Alb, known to be endemic for BoDV-1. The other two foxes (#11 and #40) originated from non-endemic regions. NGS did not detect bornavirus-like sequences. No other infectious agents were obvious as cause for the encephalitis. However, NGS confirmed the canine distemper virus infection of fox #21.

## Discussion

The present study focusses on the aetiology of unclear non-suppurative encephalitis in red foxes (*Vulpes vulpes*) in different federal states of Germany (Bavaria, Baden-Wuerttemberg and Hesse) with in particular BoDV-1 as a possible cause. This was based upon the fact, that foxes are known predators of bicoloured white-toothed shrews, a confirmed reservoir of BoDV-1. In addition, foxes are highly susceptible for infections with other viruses of the order *Mononegavirales*. The recent detection of a new zoonotic bornavirus in variegated squirrels [31] enhances the necessity to investigate for the presence of so far unknown agents such as VSBV-1, also in the wild, and in potential contact animals in particular.

Anthropogenic food resources play a big role in the diet of foxes living near human settlements. Therefore, population densities of foxes increase due to their opportunistic character and loss of their natural habitats [11]. This is why public health issues in general demand further research on the causes of non-suppurative encephalitis in animals such as foxes, as they can easily get into contact with humans, farm and pet animals. Red foxes mainly feed on abundant rodents, but in times of food shortages, they also feed on insectivores. Shrew remains in faeces of the red fox have regularly been found [48, 49]. BoDV-1 positive *C. leucodon* shrews shed high amounts of virus via skin and excretions [29] so that contact of foxes with BoDV-1 during predation is likely. The susceptibility of predators to infectious agents carried by small mammals such as BoDV-1 remains largely unknown. In principle, foxes could be resistant, develop only specific antibodies as sign of exposure, could act as spill over or accidental host, or they could become a new reservoir species. Typically, in reservoirs, the infection is clinically inconspicuous despite shedding of high amounts in virus, while in accidental hosts such as horses, sheep and recently in humans, bornavirus infection manifests itself by a strict neurotropism and progressive non-suppurative meningoencephalitis [23, 31]. Studies from experimentally infected rats revealed a T-cell mediated immunopathogenesis as key pathogenesis in diseased animals whereas in reservoir species immunotolerance mechanisms have been assumed [50–52]. This also indicates that the outcome of BoDV-1 infection differ significantly depending on the status of the immune system.

Several foxes (16/59) displayed a non-suppurative (meningo-) encephalitis highly suspicious for a viral aetiology. The hunters did not observe any neurological symptoms in 9/16 foxes with encephalitis. However, 3/16 foxes displayed signs of disease (emaciation and mange) and 4/16 foxes with encephalitis were found dead. Foxes with encephalitis originated from all three administrative districts included in the study and did therefore not follow any geographical link. Further studies in larger cohorts will have to address whether there might be hotspots for fox encephalitis in Germany. The German national rabies legislation prescribes that hunters have to shoot foxes with signs of disease or with abnormal behaviour. In addition, hunters bring more frequently carcasses of wild mammals to the veterinary inspection offices when they were found dead without signs of external trauma. Bias caused by these factors probably increased the probability to find foxes with encephalitis in this study.

By IIFT, 16.4 % of the red foxes displayed anti-BoDV-1 antibodies. Western blot analysis of 8/37 seropositive foxes confirmed the specificity of the anti-BoDV-1 antibodies. In general, endemic areas are defined by the constant presence of diseases or infectious agents in a geographic area or a population group [53]. There are no official data on endemic areas for BoDV-1 infection in Baden-Wuerttemberg, since there is no reporting obligation and no long-term data are available. However, the Swabian Alb in Baden Wuerttemberg has already been regarded as endemic area [54]. For Bavaria, more data on BD in horses are available, due to a reporting obligation up until 2011 [55]. Due to limited sample number in the present study, the statistical comparison of seropositive foxes from endemic and non-endemic areas could only serve as a rough and global comparison providing general trends. Interestingly, the presence of BoDV-1 specific serum antibodies in foxes did not correlate with their origin from endemic areas, represented by a no significant difference between detection of seropositive foxes in endemic and non-endemic areas (*p* > 0.05).

The administrative district of Swabia in Bavaria is endemic for BoDV-1 infection containing populations of BoDV-1 shedding *C. leucodon* [27, 29]. In Swabia, seven red foxes were positive for anti-BoDV-1 antibodies. At the Swabian Alb in Baden-Wuerttemberg, four foxes were seropositive. However, foxes with anti-BoDV-1 antibodies were also detected in non-endemic regions. In Hesse, 7/31 foxes displayed anti-BoDV-1 antibodies but no BD was reported in horses in the last decades in the respective areas. Interestingly, BoDV-1-infected rabbits were found in the last decades in one region (personal communication S. Herzog). Studies on the presence of reservoirs are lacking for these districts. However, Dürrwald et al. [28] also report presence of BoDV-1 positive *C. leucodon* in endemic regions in eastern Germany without equine BD cases in the last decades. In the present study, titres of seropositive foxes in the endemic regions ranged from 1:40 to 1:2650, the highest titre was found in fox #21 originating from the Swabian Alb. Another canine, a female badger from Baden-Wuerttemberg, was also positive for anti-BoDV-1 antibodies with a titre of 1:160 and did not exhibit encephalitis (data not shown).

Among six foxes with confirmed CDV-infection, five displayed additional anti-BoDV-1 antibodies. Cross reaction with antibodies against CDV is highly unlikely since specificity of anti-bornavirus antibodies was confirmed by Western blot. Therefore, a coincidental connection between CDV and BoDV-1 could be assumed. CDV is a highly immunosuppressive agent, causing lymphocyte loss and leucopenia in the acute stage [56], thereby increasing the susceptibility for opportunistic infections, e.g. for toxoplasmosis [57]. Therefore, further investigations on co-infections with BoDV-1 are necessary.

No BoDV-1 antigen (N, P) was found by IHC in the brain of the foxes with encephalitis. The combination of detection of two most abundantly expressed viral proteins ensure the detection even of BoDV-1 variants, e.g. mutations in the N gene or other bornaviruses. The fox #21 with the highest anti-BoDV-1 antibody titre was further investigated by in situ hybridization for detection of BoDV-1 RNA (according to established protocols [40]), but no genomic or mRNA was detected in the brain (data not shown). BoDV-1 RNA was not amplificated by PCR in the brain tissue by any of the applied PCR assays. In addition to the usual PCR approaches (real time RT-PCR and nested PCR), a newly developed pan-bornavirus-RT-PCR for a rapid, sensitive and economic screening of all known bornaviruses was used. As there is no knowledge on the duration of potential infection, the explanation for negative PCR results could be a low level viral persistence or also presence of virus only in specific brain areas not sampled. Moreover, even though an abundantly expressed housekeeping gene was amplificated, the sample quality and differences in sample storage could have interfered with amplification of viral sequences.

Histopathology strongly suggested a viral aetiology of the red fox encephalitis. Beside BoDV-1 [18], viral causes of encephalitis in canines are rabies virus [12], canine distemper virus (CDV) [13, 14], canine adenovirus 1 (CAV-1) [9], porcine herpesvirus 1 (SHV-1), canine parvovirus (CPV) [5, 46], West Nile virus (WNV) [5] or tick-borne encephalitis virus (TBEV) [58]. A fox circovirus [19] and La Crosse virus [17] have also been detected in canine encephalitis cases. Schwab et al. [5] found canine parainfluenza virus (CPIV) antigen in the brain of one dog and encephalomyocarditis virus (EMCV) in four dogs with encephalitis. *Toxoplasma gondii* [21], *Neospora caninum* [21] and *Encephalitozoon cuniculi* can also occasionally cause non-suppurative encephalitis. Widén et al. [15] demonstrated an up to now unclassified α-herpesvirus in arctic foxes with unclear encephalitis.

Among 16 cases of non-suppurative encephalitis in this study, six were due to CDV as confirmed by detection of viral RNA and antigen. One CDV infection (fox #21) was confirmed by metagenomic analysis. All red foxes affected by canine distemper originated from Baden-Wuerttemberg and substantiated the fact that canine distemper is a well-known infection in wild carnivores in Germany [13, 59]. All foxes were negative for rabies virus, CAV-1, SHV-1, CPV and *Toxoplasma gondii*. In addition, no flavivirus-RNA was amplified, so that WNV or TBEV infection seem unlikely.

Interestingly, no infectious agents were found for 10/16 foxes with non-suppurative (meningo-) encephalitis. Considering the retrospective nature of the study, the collected brain samples consisted of hippocampal areas for rabies virus investigation, some of them with additional presence of cerebral cortex, thalamus and meninges. Therefore, infections with agents with a certain tropism might have been missed. Several other possible causes of fox encephalitis have not been addressed in this study and could be object for further investigations, e.g. CPIV, EMCV, *Neospora caninum, Encephalitozoon cuniculi* or La Crosse virus which, however, occurs so far only in the United States. Widén et al. [15] found a herpesvirus in arctic foxes with necrotizing encephalitis. Bexton et al. [19] were the first to describe encephalitis probably due to circovirus infection in foxes in England. To date, it remains unclear if circovirus infections are a definite cause for encephalitis or if they act as contributory complicating factors, as in pigs. Recently, a certain number of unclear encephalitis in mammals have been resolved due to metagenomics [31, 60]. However, in the present study even with the metagenomic analysis, carried out for three characteristic samples with encephalitis, it was not possible to detect an infectious agent. Therefore, it could be possible that non-infectious causes of encephalitis play an important role in foxes, as known for other canines such as dogs where non-infectious encephalitis could represent a breed-specific condition [6]. Furthermore, several idiopathic conditions are known in dogs, e.g. granulomatous meningoencephalitis [22] and recently, Pruess et al. [61] reported encephalitis due to autoantibodies, a non-human anti-NMDA receptor encephalitis in a polar bear. Immune-mediated non-suppurative encephalitis has already been suspected in several cases of unclear encephalitis cases and several authors suggest that they might occur primarily or result from a virally triggered process [5, 6].

## Conclusions

Several common pathogens causing encephalitis in foxes have been taken into account in this study and a metagenomic analysis extended the ubiquitous applied methodologies. Nevertheless, in 10/16 foxes the cause for encephalitis remained unclear. Concerning bornaviruses, foxes can exhibit BoDV-1 specific serum antibodies without any further evidence of infection and seroprevalence of BoDV-1-specific antibodies in foxes was determined for the first time. In total, 37/225 red foxes exhibited anti-BoDV-1 antibodies in endemic and non-endemic regions in Germany. Thus, foxes can have contact exposure and most likely undergo abortive infection with seroconversion only and do not serve as reservoir for BoDV-1. Canine distemper virus caused non-suppurative encephalitis in 6/16 foxes but no further pathogens were found in the other foxes with unclear encephalitis. Thus, either so far unknown infectious agents, most likely a virus, or non-infectious causes have to be considered as causes fox encephalitis in the investigated districts in Germany. Non-infectious causes could also play a more important role than expected. This study is intended as a base for standardized large-scaled investigations for further clarification on fox encephalitis.
